# Simulating of effective conductivity for graphene–polymer nanocomposites

**DOI:** 10.1038/s41598-023-32991-w

**Published:** 2023-04-11

**Authors:** Mostafa Vatani, Yasser Zare, Nima Gharib, Kyong Yop Rhee, Soo-Jin Park

**Affiliations:** 1 Department of Chemical Engineering, Faculty of Engineering, University of Kashan, P.O. Box 87317-53153, Kashan, Iran; 2 Biomaterials and Tissue Engineering Research Group, Department of Interdisciplinary Technologies, Breast Cancer Research Center, Motamed Cancer Institute, ACECR, Tehran, Iran; 3 College of Engineering and Technology, American University of the Middle East, Egaila, 54200 Kuwait; 4 Department of Mechanical Engineering (BK21 Four), College of Engineering, Kyung Hee University, Yongin, Republic of Korea; 5 Department of Chemistry, Inha University, Incheon, 22212 Republic of Korea

**Keywords:** Engineering, Materials science

## Abstract

The efficient conductivity of graphene-polymer systems is expressed supposing graphene, tunneling and interphase components. The volume shares and inherent resistances of the mentioned components are used to define the efficient conductivity. Besides, the percolation start and the share of graphene and interphase pieces in the nets are formulated by simple equations. Also, the resistances of tunneling and interphase parts are correlated to graphene conductivity and their specifications. Suitable arrangements among experimented data and model’s estimates as well as the proper trends between efficient conductivity and model’s parameters validate the correctness of the novel model. The calculations disclose that the efficient conductivity improves by low percolation level, dense interphase, short tunnel, large tunneling pieces and poor polymer tunnel resistivity. Furthermore, only the tunneling resistance can govern the electron transportation between nanosheets and efficient conductivity, while the big amounts of graphene and interphase conductivity cannot play a role in the efficient conductivity.

## Introduction

Graphene has a high stiffness and good electrical conductivity causing the stiff and conductive nanocomposites^1–10^. The early studies on polymer graphene nanocomposites have focused on the preparation of products with small percolation start to achieve a big conductivity by a slight filler amount^11–13^. Generally, the percolation start is linked to the aspect ratio of graphene as the ratio of diameter to thickness^14,15^. So, the dimensions, dispersion quality and aggregation/agglomeration of graphene nanosheets manage the percolation start and thus, the nanocomposite’s conductivity (denoted as conductivity here). In addition, some novel parameters attributed to nanoscale including tunneling effect and interphase area can control the percolation start and conductivity.

**Figure 1:**
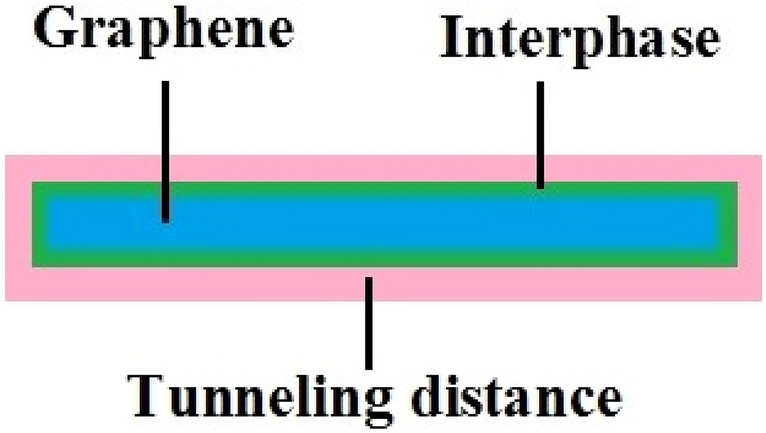
Schematic of graphene, interphase and tunneling regions in a nanocomposite.

The tunneling mechanism mainly governs the conductivity^16–19^. In fact, electrons can be easily transported via tunnels between nanoparticles. Therefore, the conductivity does not require the physical joining of nanoparticles. Figure 1 shows the tunneling space around nanoparticles by a schematic. Also, the tunneling effect changes the levels of percolation start and conductivity. However, only scarce studies have engrossed on the tunneling conductivity in CNT-based system. Moreover, the interphase pieces caused by the huge superficial area of nanoparticles efficiently affect the mechanical behavior of nanocomposites^20–25^. Figure 1 shows the interphase around the graphene in a nanocomposite. Many modeling techniques were developed to express the importance of interphase features on the robustness of nanocomposites^26–29^. The interphase pieces can also connect to the nets, since they cover the nanoparticles^30,31^. This attractive subject have been studied for rigidity of nanocomposites^32,33^. Nevertheless, the impress of interphase on the conductivity was improperly studied.

Some novel models were suggested for conductivity of CNT-based nanocomposites^19,34,35^. They have assumed the roles of many terms for CNT such as amount, conductivity, waviness and aspect ratio in the conductivity. Also, some limited studies have exposed the significances of tunneling effect and interphase on the conductivity of CNT products, but their complex analysis limits their application in practice^36–38^. The theoretic works on the conductivity of graphene samples are also imperfect. The former authors commonly correlated the percolation start to aspect ratio and estimated the conductivity by old models such as power-law^39–41^. However, the interphase pieces and tunneling largely affect the percolation start and conductivity of polymer graphene nanocomposites. Obviously, disregarding of these main terms in the nanocomposites cannot lead to proper prediction of their behavior. Therefore, development of new models for calculation of conductivity considering these terms are valuable.

In our previous work^42^, a model for conductivity of graphene systems was proposed by interphase depth, filler network, network efficiency and tunneling properties. In the present work, the efficient conductivity of graphene-filled products is expressed assuming the volume shares and resistances of graphene, tunneling and interphase parts. Also, the percolation start and the shares of graphene and interphase pieces in the nets are stated by simple equations. The percolation start and the dimensions of graphene, tunneling and interphase parts express the volume shares of these parts in the nets. Additionally, the resistance of interphase piece is correlated to filler conductivity and interphase deepness. The innovative model is justified by the tried data of some examples. In addition, the impresses of factors on the efficient conductivity are explained and discussed.

## Theoretical procedure

The actual resistance of polymer graphene nanocomposites was expressed^43^ as:

1 $$\rho_{eff} = \frac{2tR}{{\varphi_{f} }},$$

where “t” is graphene thickness, “$\varphi_{f}$” is filler amount and “R” is the inherent resistance of sample.

**Figure 2:**
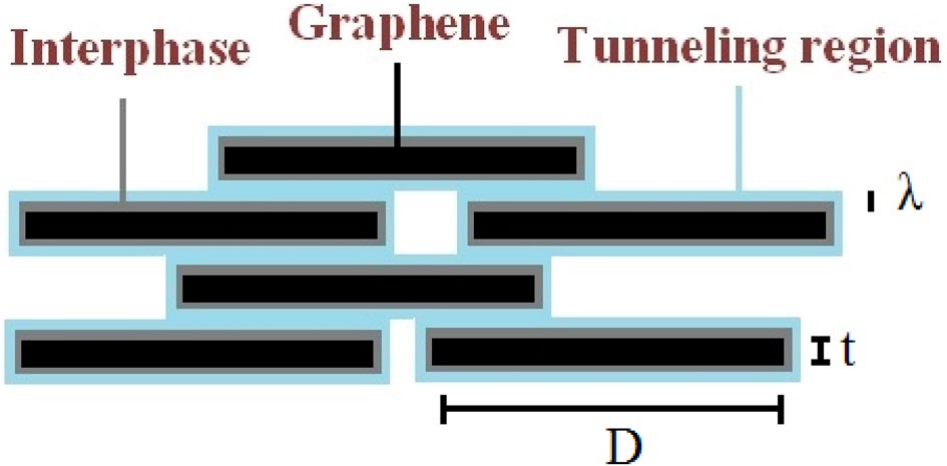
Graphene nanosheets, tunneling and interphase pieces in nanocomposites.

The polymer nanocomposites include the filler, tunneling and interphase pieces, which affect the efficient conductivity. Figure 2 displays the mentioned components and their dimensions in the nanocomposites. As observed, the interphase pieces covet the nanoparticles and the tunneling pieces form between adjacent nanosheets.

The latter equation can consider the size, resistance and volume share of each component in nanocomposites as:

2 $$\rho_{eff} = \frac{{2tR_{f} }}{{\varphi_{f} }} + \frac{{2t_{i} R_{i} }}{{\varphi_{i} }} + \frac{{2\lambda R_{t} }}{{\varphi_{t} }},$$

where subscripts “f”, “t” and “i” show the filler, tunnels and interphase, correspondingly. Also, “t_i_” and “λ” are interphase deepness and tunneling length, correspondingly.

Since only the shares of filler, tunneling and interphase pieces in the nets can affect the actual resistance, the latter equation is expressed as:

3 $$\rho_{eff} = \frac{{2tR_{f} }}{{\varphi_{N} }} + \frac{{2t_{i} R_{i} }}{{\varphi_{iN} }} + \frac{{2\lambda R_{t} }}{{\varphi_{tN} }},$$

where “$\varphi_{N}$”, “$\varphi_{iN}$” and “$\varphi_{tN}$” are the volume shares of filler, tunneling and interphase zones in the conductive nets.

The efficient conductivity can be suggested by inverse “$\rho_{eff}$” as:

4 $$\sigma_{eff} = \frac{1}{{\frac{{2tR_{f} }}{{\varphi_{N} }} + \frac{{2t_{i} R_{i} }}{{\varphi_{iN} }} + \frac{{2\lambda R_{t} }}{{\varphi_{tN} }}}}.$$

All parameters should be determinate to calculate the efficient conductivity.

The percolation start in polymer graphite nanocomposites was stated^15^ by:

5 $$\varphi_{p} = \frac{{27\pi D^{2} t}}{{4(D + \lambda )^{3} }},$$

where “D” is the graphene diameter.

Since D >  > λ, this equation is simplified to:

6 $$\varphi_{p} = \frac{27\pi t}{{4D}}.$$

The latter equation can be developed assuming tunneling and interphase pieces as:

7 $$\varphi_{p} = \frac{{27\pi t^{2} }}{{4Dt + 2(Dt_{i} + D\lambda )}}.$$

So, the percolation start can be estimated by filler size, interphase deepness and tunneling length.

The interphase pieces also promote the efficiency of nanofillers in nanocomposites. The total volume portion of interphase in polymer graphene nanocomposites^44^ is given by:

8 $$\varphi_{i} = \varphi_{f} \left( {\frac{{2t_{i} }}{t}} \right).$$

Since the interphase pieces can add to filler nets, the actual volume share of nanofiller consists of both graphene and interphase shares as:

9 $$\varphi_{eff} = \varphi_{f} + \varphi_{i} = \varphi_{f} \left( {1 + \frac{{2t_{i} }}{t}} \right).$$

Also, the percentages of nanosheets contributing to the conductive nets^45^ can be calculated by:

10 $$f = \frac{{\varphi_{f}^{1/3} - \varphi_{p}^{1/3} }}{{1 - \varphi_{p}^{1/3} }}.$$

Supposing the contribution of interphase pieces to the conductive nets by “$\varphi_{eff}$” (Eq. 9) and “$\varphi_{p}$” (Eq. 7), “f” is developed to:

11 $$f = \frac{{\varphi_{eff}^{1/3} - \varphi_{p}^{1/3} }}{{1 - \varphi_{p}^{1/3} }},$$

expressing the share of both graphene and interphase parts in the nets.

So, the volume share of networked graphene is calculated by:

12 $$\varphi_{N}^{{}} = f\varphi_{f}^{{}} ,$$

“f” can be substituted form Eq. (11), which suggests:

13 $$\varphi_{N}^{{}} = \frac{{\varphi_{eff}^{1/3} - \varphi_{p}^{1/3} }}{{1 - \varphi_{p}^{1/3} }}\varphi_{f}^{{}} .$$

Furthermore, the volume share of interphase pieces in the nets is given by:

14 $$\varphi_{iN}^{{}} = f\varphi_{i}^{{}} .$$

When “f” (Eq. 11) and “$\varphi_{i}^{{}}$” (Eq. 8) are exchanged into above equation, “$\varphi_{iN}^{{}}$” is expressed by:

15 $$\varphi_{iN}^{{}} = \frac{{\varphi_{eff}^{1/3} - \varphi_{p}^{1/3} }}{{1 - \varphi_{p}^{1/3} }}\varphi_{f} \left( {\frac{{2t_{i} }}{t}} \right).$$

Also, it can be suggested that the interfacial attachments manipulate the interphase conductivity in nanocomposites. The interphase deepness commonly represents the extent of interfacial attachments. Consequently, the interphase conductivity is related to interphase deepness as:

16 $$\sigma_{i} = \frac{{t_{i} \sigma_{f} }}{{t_{m} }},$$

where “t_m_” is top interphase deepness in graphene nanocomposites. “t_m_” is 40 nm in the present study. This equation recommends the interphase conductivity by interphase deepness and filler conductivity.

Moreover, the inherent resistances of graphene and interphase parts can be expressed by:

17 $$R_{f} = \frac{D}{{\sigma_{f} Dt}} = \frac{1}{{\sigma_{f} t}},$$

18 $$R_{i} = \frac{D}{{\sigma_{i} Dt_{i} }} = \frac{{t_{m} }}{{t_{i}^{2} \sigma_{f} }}.$$

Equations (13), (15), (17) and (18) determine the volume shares and inherent resistances of graphene and interphase pieces in the nets, which can be substituted in Eq. (4) to estimate the efficient conductivity.

Now, the volume share and inherent resistance of tunneling pieces are defined.

It was found that the graphene nanosheets are overlapped in nanocomposites and the tunneling conductivity occurs by overlapped nanosheets (Fig. 2)^43^.

The total volume share of tunnels around interphase part in nanocomposites can be given like Eq. (8) by:

19 $$\varphi_{t} = (\varphi_{f} + \varphi_{i} )\left( {\frac{2\lambda }{{t + 2t_{i} }}} \right).$$

But, only the tunnels in the nets are operative on the efficient conductivity. So, the share of tunnels in the nets is assumed as:

20 $$\varphi_{tN} = (\varphi_{N} + \varphi_{iN} )\left( {\frac{2\lambda }{{t + 2t_{i} }}} \right).$$

The tunneling pieces in nanocomposites contain polymer layer and graphene nanosheets. As a result, the tunneling resistance considers the inherent resistance of graphene nanosheets in tunneling zones (R_g_) and the polymer tunnel resistivity (R_p_) as:

21 $$R_{t} = R_{g} + R_{p} .$$

“R_g_” and “R_p_” can be suggested^43^ by:

22 $$R_{g} = \frac{1}{{\sigma_{f} d}},$$

23 $$R_{p} = \frac{\rho \lambda }{S} = \frac{\rho \lambda }{{d^{2} }},$$

where “d” shows the tunneling diameter, “ρ” is polymer tunnel resistivity and “S” denotes the tunneling area (S ≈ d^2^).

Assuming Eqs. (21–23), the inherent resistance of tunnels is defined by:

24 $$R_{t} = \frac{1}{{\sigma_{f} d}} + \frac{\rho \lambda }{{d^{2} }},$$

expressing that the inherent resistance of tunnels links to the graphene conductivity, tunneling length, tunneling area and the polymer tunnel resistivity.

When “$\varphi_{N}^{{}}$” (Eq. 13), “R_f_” (Eq. 17), “$\varphi_{iN}^{{}}$” (Eq. 15), “R_i_” (Eq. 18), “$\varphi_{tN}^{{}}$” (Eq. 20) and R_t_ (Eq. 24) are substituted in Eq. (4), the efficient conductivity is presented by the physical appearances of graphene, tunneling and interphase parts. The novel model can clearly determine the significances of each parameter in the efficient conductivity.

## Results and discussion

### Comparison of model with experimented facts

The novel model is used to calculate the efficient conductivity in several examples from published articles. Several graphene examples including polyimide (PI) ($\varphi_{p}^{{}}$ = 0.0015, D = 5 μm, t = 3 nm) from Ref.^46^, polystyrene (PS) ($\varphi_{p}^{{}}$ = 0.0005, D = 4 μm, t = 1 nm) from Ref.^47^, poly (ethylene terephthalate) (PET) ($\varphi_{p}^{{}}$ = 0.005, D = 2 μm, t = 2 nm) from Ref.^48^ and styrene acrylonitrile (SAN) ($\varphi_{p}^{{}}$ = 0.0017, D = 2 μm, t = 1 nm) from Ref.^49^ were chosen. The application of novel model needs the determination of all factors for tunneling and interphase pieces. Use of Eq. (7) for percolation start can guess the average values of interphase deepness and tunneling length. The (t_i_, λ) are calculated as (30, 12), (7, 10), (5, 8) and (6, 5) nm for PI, PS, PET and SAN nanocomposites, correspondingly. These levels demonstrate the formation of large interphase and big tunnels in the examples. So, it can be concluded that both interphase deepness and tunneling length control the percolation start and efficient conductivity. These calculations are applied in the novel model to guess the efficient conductivity.

**Figure 3:**
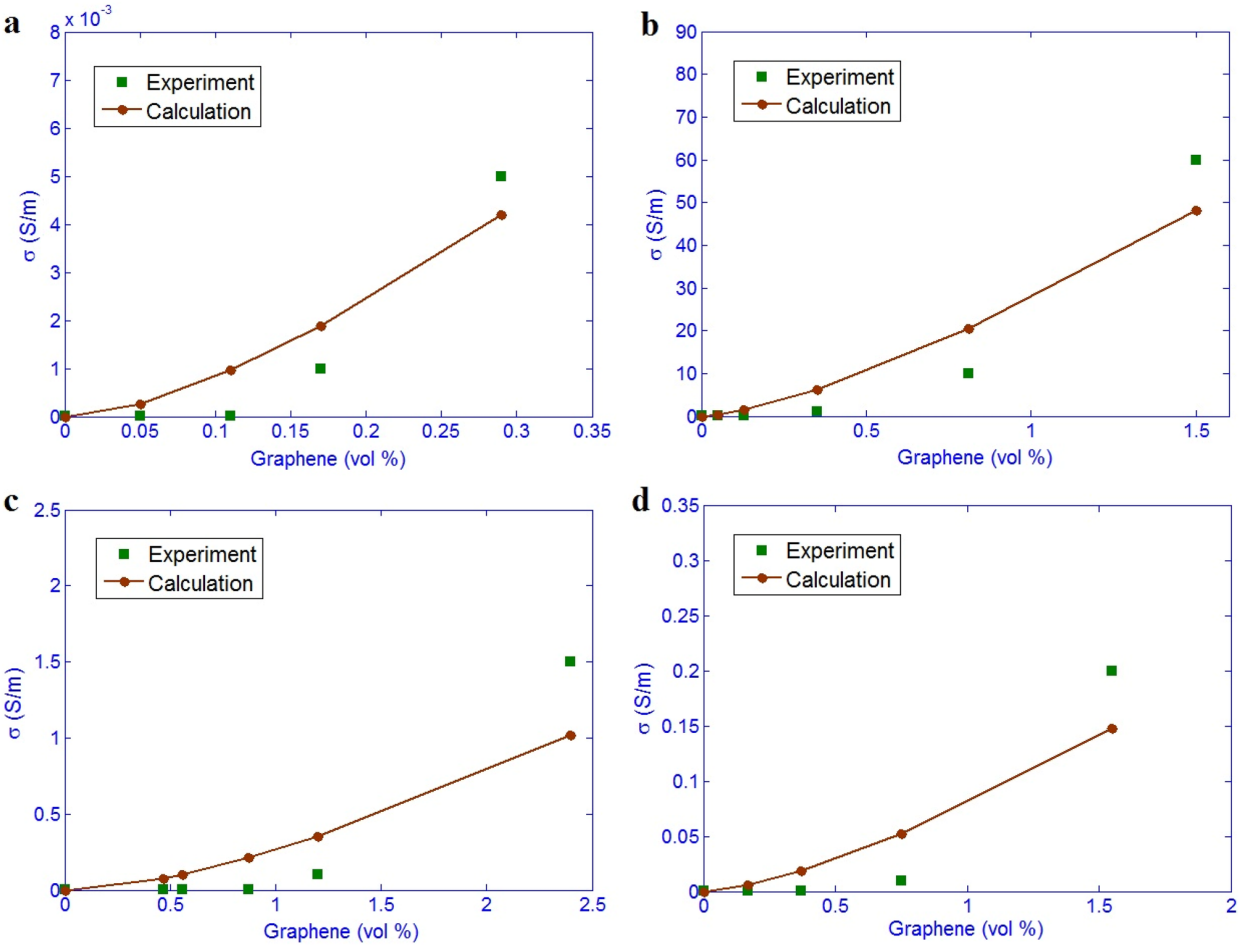
Relationship between experimented and theoretical results for (**a**) PI/graphene from Ref.^46^, (**b**) PS/graphene from Ref.^47^, (**c**) PET/graphene from Ref.^48^ and (**d**) SAN/graphene from Ref.^49^.

Figure 3 exemplifies the experimented quantities and the predicted amounts of efficient conductivity for the examples. The forecasts acceptably follow the tested values at different filler amounts. Therefore, the novel model shows a good predictability assuming graphene, tunneling and interphase components.

The extents of (d, ρ) are obtained as (200, 240), (2000, 60), (400, 100) and (300, 1000) (nm, ohm.m) for PI, PS, PET and SAN graphene examples, correspondingly. The highest “d” and the lowest “ρ” are shown in PS/graphene nanocomposite, which cause the minimum level of inherent resistance of tunnels (Eq. 24). So, it is expected that this sample shows the highest effective conductivity. Figure 3 demonstrates that the PS/graphene sample shows the highest efficient conductivity among the examples. Consequently, the efficient conductivity mainly depends on the tunneling properties, because the tunneling effect is the main mechanism of electrical conductivity in polymer nanocomposites. There are some errors between experimental data and our calculations in Fig. 3, but this is acceptable from theoretical point of view, because it is lower than 10%. Actually, low difference (maximum 10%) between experimental data and calculations are acceptable in the modeling studies. It is believed that some terms such as aggregation/agglomeration of nanoparticles may produce the error, which should be removed from the samples.

### Examination of parameters

The guesstimates of the advanced model at altered series of all factors can be plotted and evaluated. By these analyses, it is possible to show the significance of each parameter on the efficient conductivity. 3D and contour plans are used to exhibit the variations of efficient conductivity at many series of two factors. The mediocre points of parameters in all calculations are considered as D = 2 μm, $\varphi_{f}$ = 0.01, t_i_ = 5 nm, t = 2 nm, λ = 5 nm, σ_f_ = 10^5^ S/m, d = 300 nm and ρ = 500 Ω m.

**Figure 4:**
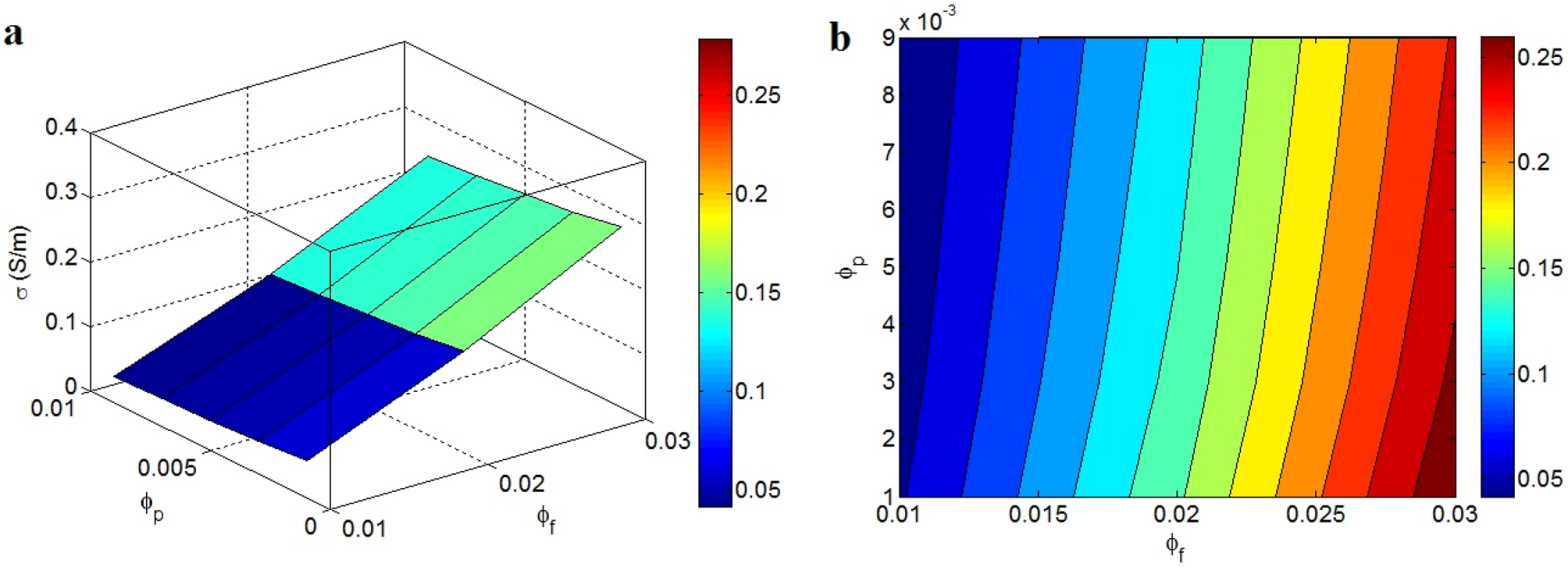
The predictions of novel model at various ranks of “$\varphi_{f}$” and “$\varphi_{p}$”: (**a**) 3D and (**b**) contour configurations.

Figure 4 reveals the efficient conductivity at the changed points of “$\varphi_{f}$” and “$\varphi_{p}$”. The biggest efficient conductivity of 0.026 S/m is obtained by $\varphi_{f}$ = 0.03 and $\varphi_{p}$ = 0.001, while $\varphi_{f}$ = 0.01 produces the efficient conductivity of about 0.05 S/m. These evidences disclose that the efficient conductivity recovers by big filler quantity and low percolation level, but slight filler amount cannot considerably enhance the efficient conductivity. Obviously, a big quantity of conductive nanoparticles produces the huge nets, which can significantly improve the efficient conductivity. In fact, the dimension and compactness of nets mostly depend on the amount of nanoparticles in the products. However, a low amount of nanoparticles cannot produce large nets deteriorating the conductivity, because the small nets cannot efficiently transfer the charges. Therefore, it is evident that the amount of graphene directly affects the efficient conductivity. Additionally, a low percolation start can provide the conductivity by little filler amount. Also, big nets can be made by low percolation start (Eq. 11). Thus, the conductivity inversely relates to the percolation start. By this explanation, the novel model acceptably states the impacts of “$\varphi_{f}$” and “$\varphi_{p}$” on the efficient conductivity.

**Figure 5:**
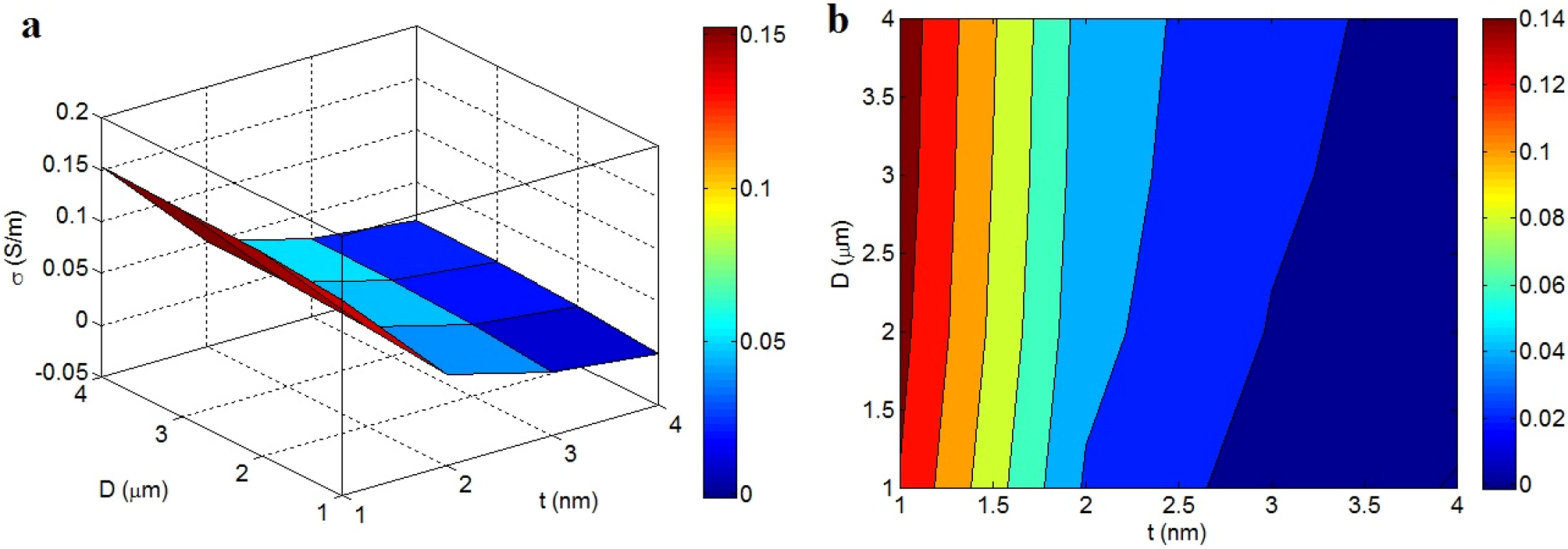
Variations of efficient conductivity at various points of “t” and “D”: (**a**) 3D and (**b**) contour designs.

Figure 5 illustrates the impacts of “t” and “D” on the efficient conductivity. The efficient conductivity reaches to 0.14 S/m at t = 1 nm and D > 1.5 μm, nevertheless the efficient conductivity significantly weakens to 0 at t > 3.5 nm or t > 3 and D < 2 μm. Accordingly, thin and big nanosheets desirably affect the efficient conductivity, but thick and short graphene cannot improve it. Also, it is observed that the dimensions of graphene considerably control the efficient conductivity.

A poor percolation start is obtained by thin and big nanosheets, which upsurges the extent of nets. Also, the interphase pieces enlarge by thin graphene based on Eq. (15). In other words, thin and big nanosheets positively manipulate the percolation start and interphase pieces that promote the magnitude of conductive nets in nanocomposites. So, it is expected that the efficient conductivity improves by thin and big nanosheets. The impresses of these factors on the percolation start have been suggested in earlier works^50,51^, but their effects on the efficient conductivity have not been reported, yet.

**Figure 6:**
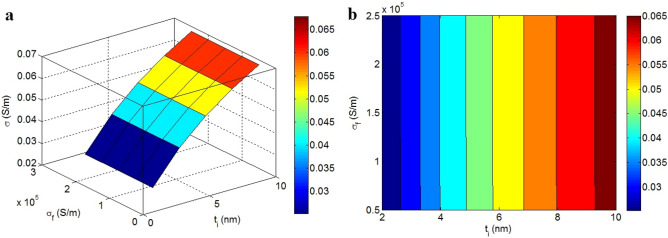
The efficient conductivity by the changes in “t_i_” and “σ_f_”: (**a**) 3D and (**b**) contour schemes.

Figure 6 portrays the impacts of “t_i_” and “σ_f_” on the efficient conductivity. “t_i_” only changes the efficient conductivity, although various points of “σ_f_” cannot manage the efficient conductivity. The “t_i_” value of 10 nm produces the efficient conductivity of 0.065 S/m, however the efficient conductivity of 0.025 S/m is observed at t_i_ = 2 nm. Thus, the size of interphase directly influences the efficient conductivity, while the filler conductivity cannot govern it.

A dense interphase considerably declines the percolation start and promotes the size of conductive nets, because both graphene and interphase pieces form the conductive nets. Moreover, a dense interphase indicating the sturdy interfacial interactions increases the conductivity of interphase layer (Eq. 16). So, a dense interphase enlarges the nets and enriches their conductivity growing the efficient conductivity. The encouraging role of interphase deepness in the conductivity of CNT system was insufficiently shown in a previous article^52,53^. Furthermore, the efficient conductivity associates to the inherent resistance of each component (Eq. 4). However, the wonderful conductivity of graphene mainly deteriorates its inherent resistance, which cannot produce a considerable opposition affecting the conductivity. Really, the negligible resistance of graphene nanosheets cannot act against the electron current^54,55^. Consequently, the model satisfactorily shows the ineffectual weight of high graphene conductivity in the efficient conductivity.

**Figure 7:**
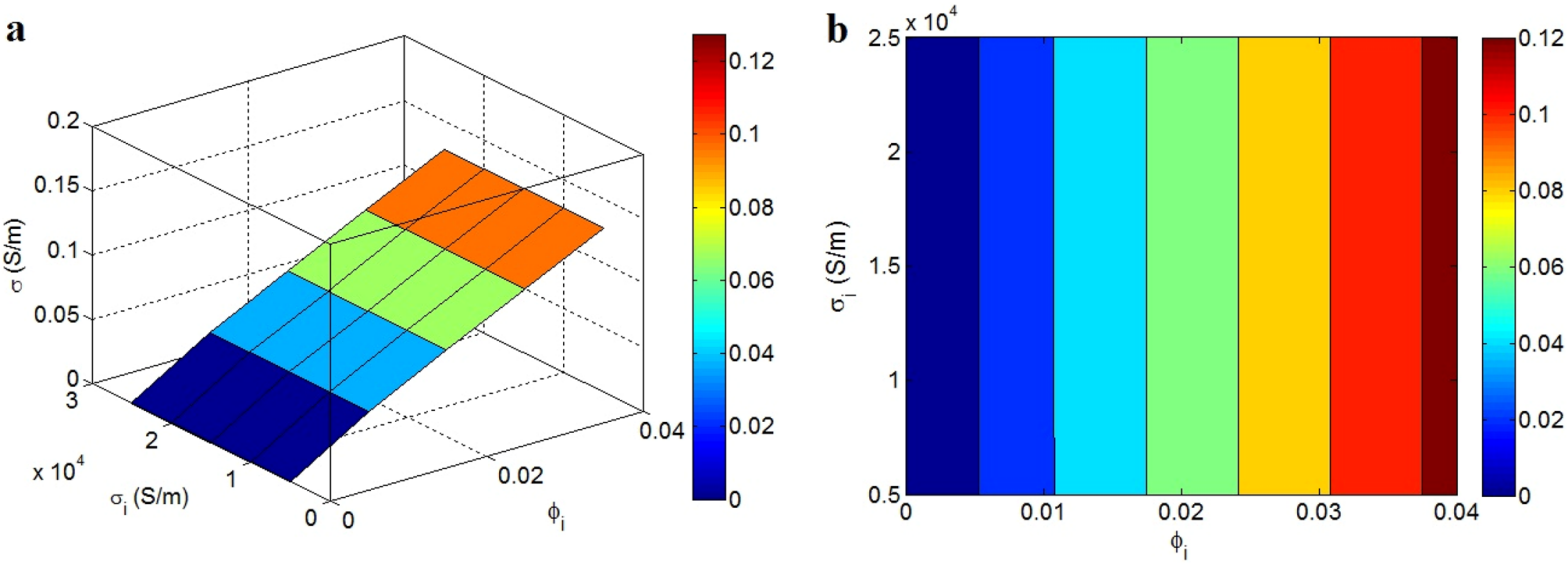
Illustration of efficient conductivity at countless points of “$\varphi_{i}$” and “σ_i_”: (**a**) 3D and (**b**) contour images.

Figure 7 determines the impresses of “$\varphi_{i}$” and “σ_i_” on the efficient conductivity. The maximum $\varphi_{i}$ = 0.04 harvests the efficient conductivity of 0.12 S/m, although $\varphi_{i}$ < 0.005 cannot improve the efficient conductivity. In addition, “σ_i_” does not change the efficient conductivity. It can be stated that the interphase amount straightly handles the efficient conductivity, but the interphase conductivity cannot deploy it. In fact, the large interphase piece is necessary to produce a desirable conductivity.

The interphase pieces reduce the percolation start and expand the conductive nets. Also, the conductivity of interphase pieces is more than that of polymer matrix. So, big interphase pieces can harvest the efficient nets in the nanocomposites, which encourage the high efficient conductivity. Oppositely, small interphase pieces cannot manipulate the percolation start and network structures, which negligibly change the efficient conductivity. Also, it was mentioned that the high-resistant components in nanocomposites can disturb the efficient conductivity. However, the high range of interphase conductivity deteriorates its resistance. Actually, the interphase conductivity cannot influence the efficient conductivity, because it cannot produce a significant resistance against the transportation of electrons in nanocomposites. By these descriptions, the novel model precisely foresees the meanings of interphase deepness and conductivity on the efficient conductivity.

**Figure 8:**
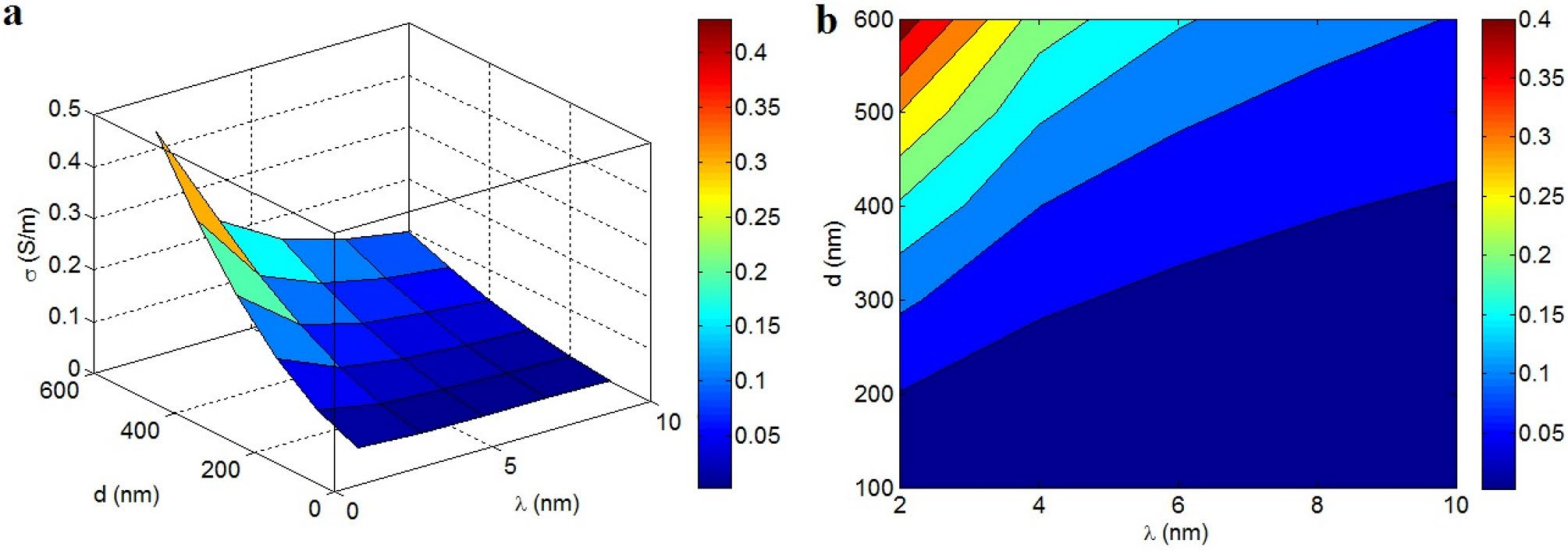
Expression of efficient conductivity at various points of “λ” and “d” by (**a**) 3D and (**b**) contour patterns.

Figure 8 exposes the importance of “λ” and “d” on the efficient conductivity. The top efficient conductivity of 0.4 S/m is found by λ = 2 nm and d = 600 nm, but the efficient conductivity decreases to 0 at d < 200 nm or λ > 8 nm and d < 400 nm. Accordingly, large tunnels and poor tunneling diameter weaken the efficient conductivity, but short tunnels and large tunneling area can advance the efficient conductivity.

A large tunnel grows the volume of polymer in the tunnels. Indeed, a large tunnel includes the dense polymer film enhancing the contact resistance. As a result, a large tunnel worsens the efficient conductivity, owing to the weakness of electron transportation in tunneling pieces. Alternatively, a high tunneling diameter demonstrates that the large graphene nanosheets cover the tunneling pieces. So, a big tunneling diameter deteriorates the contact resistance and facilitates the electron transportation through tunneling pieces. These observations justify the negative and positive significances of large tunnels and big tunneling area on the efficient conductivity, as recommended by the novel model.

**Figure 9:**
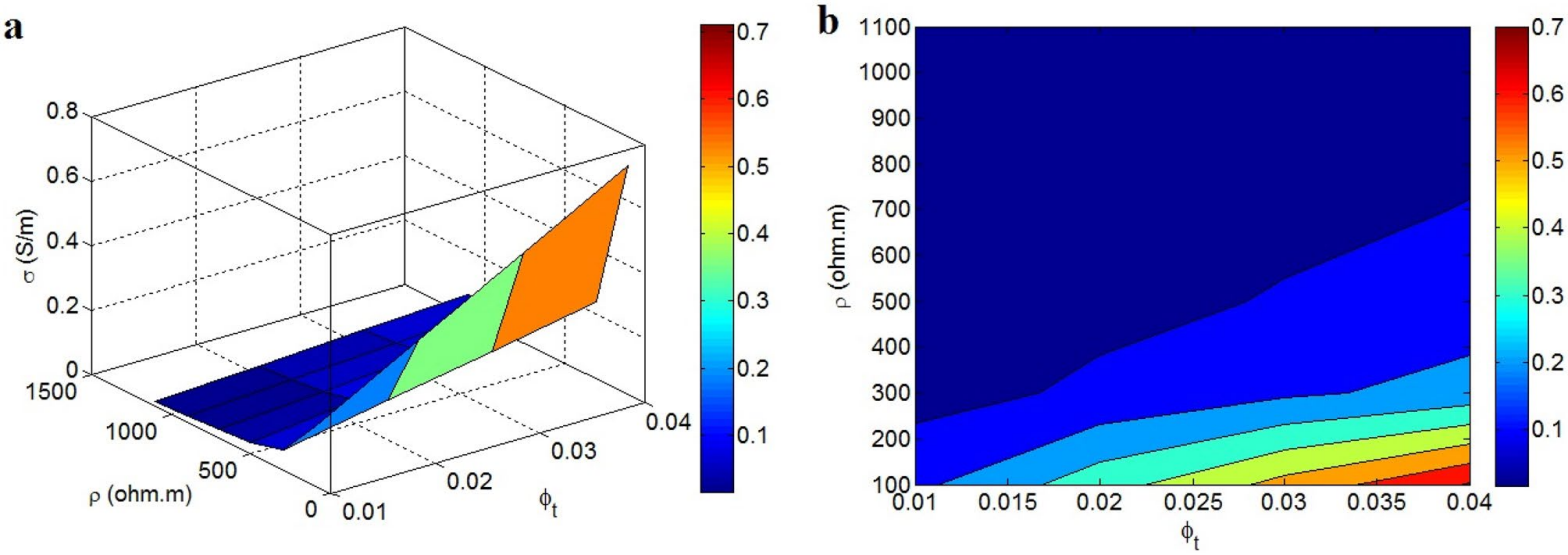
(**a**) 3D and (**b**) contour plans for the impresses of “$\varphi_{t}$” and “ρ” on the efficient conductivity.

Figure 9 expresses the linking of efficient conductivity to “$\varphi_{t}$” and “ρ”. High “$\varphi_{t}$” and poor “ρ” desirably affect the efficient conductivity, nevertheless the big values of “ρ” result in an insulated nanocomposite. It can be suggested larger contact pieces and lower polymer tunnel resistivity produce a higher efficient conductivity, while small tunneling zones with high tunnel resistivity significantly reduce it. Indeed, the efficient conductivity improves by large and poor-resistant tunneling pieces.

Big tunnels efficiently decrease the percolation start and expand the nets. However, the high polymer tunnel resistivity restricts the transportation of electrons and weakens the efficient conductivity. So, it should be suggested that the formation of big and conductive tunneling pieces can raise the efficient conductivity, because they largely transfer the electrons and induce high conductivity. Instead, short tunneling zones shorten the size of nets. Additionally, the great level of polymer tunnel resistivity declines the tunneling conductivity and efficient conductivity. The novel model displays the more important role of high polymer tunnel resistivity compared to tunneling share. Since a great polymer tunnel resistivity considerably limits the electron transference in all tunneling pieces (short or large ones), the estimates of the novel model are acceptable. By these signs, the novel model fittingly displays the variations of efficient conductivity at numerous ranges of “$\varphi_{t}$” and “ρ”.

**Figure 10:**
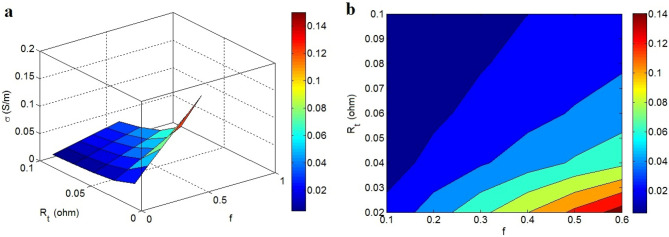
Dependences of efficient conductivity on “f” and “R_t_” by (**a**) 3D and (**b**) contour designs.

Figure 10 displays the association of efficient conductivity to “f” and “R_t_”. The maximum efficient conductivity of 0.14 S/m is shown at f = 0.6 and R_t_ = 0.02 Ω, whereas the efficient conductivity decreases to 0 at f < 0.3 and R_t_ > 0.07 Ω. These outputs designate that the efficient conductivity improves by the high shares of nanosheets and interphase in the nets as well as poor resistance of tunneling pieces. Furthermore, it is understood that the poor share of networked structures and high tunneling resistance meaningfully worsen the efficient conductivity.

The size and compactness of nets expectedly manage the efficient conductivity, for the reason that the conductive nets containing nanosheets and interphase pieces control the extent of electron shifting in nanocomposites^56–58^. The large nets provide the efficient transference of electrons, but the small nets weaken the electron moving. Thus, it is rational to get a better efficient conductivity by higher “f”. Moreover, the tunneling resistance oppositely governs the efficient conductivity, because its high level restricts the conductivity of tunneling pieces and the charge transpiration. It is also concluded that only the high resistance of tunneling pieces affects the efficient conductivity, because the negligible levels of graphene and interphase resistances cannot control the electron moving. In fact, great tunneling resistance weakens the charge flow, but graphene and interphase components cannot produce actual resistance in the system. Therefore, the tunneling resistance regulates the efficient conductivity, as proposed by the novel model.

## Conclusions

The efficient conductivity was expressed by graphene, tunneling and interphase parts. Also, the volume shares of these components in the nets and their inherent resistances were defined by simple equations. The novel model reveals good predictability compared to tested conductivity of examples. Similarly, the parametric examinations properly justify the stimuli of all factors on the efficient conductivity. These indications approve the correctness of the novel model. The predictions reveal that high filler amount, low percolation level, thin and big nanosheets, dense interphase, short tunnels, wide tunneling area and low tunnel resistivity grow the efficient conductivity. However, graphene and interphase components cannot resist against the electron transportation, while the tunneling resistance governs the electron current. Accordingly, only tunneling resistance affects the efficient conductivity. Among the studied factors, the size of tunnels and tunnel resistivity mainly affect the efficient conductivity. The efficient conductivity can grow to 0.7 S/m at $\varphi_{t}$ = 0.04 and ρ = 100 Ω m, while an insulated product is witnessed at high “ρ”.

## Data Availability

The data that support the findings of this study are available on a request from corresponding author.
